# International Expert Priorities for Promoting Recovery and Coercion-Free Practices in Psychosocial Disability: A Delphi Study

**DOI:** 10.31083/AP46500

**Published:** 2026-04-24

**Authors:** Michela Atzeni, Giulia Cossu, Massimo Tusconi, Peter Konstantin Kurotschka, Thurayya Zreik, Antonio Egidio Nardi, Marietta Khurshudyan, Goce Kalcev, Silvia Ciccu, Oye Gureje, Rym Ghachem, Cesar Ivan Aviles Gonzalez, Mauro Giovanni Carta

**Affiliations:** ^1^University Hospital of Cagliari, 09042 Cagliari, Italy; ^2^Department of Medical Sciences and Public Health, University of Cagliari, Monserrato Blocco I (CA), 09042 Cagliari, Italy; ^3^Department of General Practice, University Hospital Würzburg, 97070 Würzburg, Germany; ^4^Mental Health Service User Association, Beirut, Lebanon; ^5^Capacities Building for Global Health, University of Cagliari, 09042 Cagliari, Italy; ^6^Instituto de Psiquiatria, Universidade Federal do Rio de Janeiro, Rio de Janeiro, RJ 22290-140, Brazil; ^7^Armenian Psychiatric Association, 0025 Yerevan, Armenia; ^8^The National Alliance for Neuromuscular Diseases and Neuroscience GANGLION Skopje, 1000 Skopje, North Macedonia; ^9^Associazione Sarda per l'Attuazione della Riforma Psichiatrica (ASARP), 09127 Cagliari, Italy; ^10^Department of Psychiatry, University of Ibadan, University College Hospital, Ibadan, Nigeria; ^11^Department of Psychiatry, Stellenbosch University, Stellenbosch, South Africa; ^12^Razi Hospital, 2010 Tunis, Tunisia; ^13^Department of Medicine and Surgery, University of Kore, Piazza dell'Università, 94100 Enna, Italy; ^14^Tropical Medicine, Universidad Popular del Cesar, 200001 Valledupar, Colombia; ^15^Department of Nursing, Universidad Popular del Cesar, 200001 Valledupar, Colombia

**Keywords:** human rights, persons with disabilities, mental health recovery, coercion, Delphi study, Delphi method

## Abstract

The global crisis of recent years, including pandemics, armed conflicts, climate emergencies, and economic instability, has disproportionately jeopardized the health and rights of persons with psychosocial disabilities, who remain among the most marginalized groups. In this context, promoting a rights-based approach to mental health care is both urgent and ethically imperative. Building on the United Nations (UN) Convention on the Rights of Persons with Disabilities and the World Health Organization (WHO) QualityRights initiative, this study aimed to identify expert-informed priorities for implementing recovery-oriented, coercion-free practices in consultation-liaison psychiatry within a general hospital in Italy, while also incorporating perspectives from international stakeholders. A two-round Policy Delphi study was conducted between December 2024 and May 2025 with 17 invited experts from diverse professional and cultural backgrounds. Nine completed both rounds. The Delphi approach was used to identify areas of convergence while also capturing the diversity of expert perspectives. No fixed consensus threshold was set, reflecting the view that in ethically and contextually complex domains such as human rights in mental health, divergent opinions can yield valuable insights. In Round 1, participants provided qualitative responses to four open-ended questions, which were analyzed thematically to generate items for Round 2. In Round 2, participants prioritized structured response options (n = 8 items) derived from the thematic analysis. Descriptive statistics summarized the response distributions. Round 1 revealed strong convergence on the importance of community inclusion, user participation, and professional training. Round 2 confirmed these priorities: 78% identified ‘mutual exchange and learning' as the main benefit of international collaboration, while 44% and 33% prioritized social inclusion and staff training, respectively, as essential for rights-based recovery. Reported barriers included control-oriented care cultures (33%) and insufficient training or tools (22% each). Experts envisioned future mental health care as fully integrated into community life (56%), with reduced reliance on institutional structures or technocratic solutions. This Delphi study underscores that rights-based, recovery-oriented mental health services must prioritize community inclusion, shared governance, and contextual adaptability. Experts cautioned against top-down or standardized reforms, favoring relational and participatory models. These findings provide actionable insights for developing coercion-free consultation-liaison psychiatry within general hospitals and can inform broader international efforts toward sustainable, rights-based mental health reform.

## Main Points

1. Experts prioritized participatory, relational, and community-embedded models as the foundation of rights-based mental health care.

2. Contextual adaptation was considered essential, while standardized, top-down or technocratic reforms received little support.

3. Social inclusion consistently emerged as a key lever for promoting recovery, reducing stigma, and protecting rights.

4. Strengthening shared governance and decision-support tools was viewed as central to enhancing autonomy.

5. The envisioned future of mental health care is fully integrated into community life, with reduced reliance on institutional structures.

## 1. Introduction

In the current global context, marked by economic instability, pandemics, armed conflicts, and climate emergencies, the rights of persons with psychosocial disabilities remain critically vulnerable. During the COVID-19 pandemic, mortality among people with severe psychiatric conditions was twice as high as that of the general population [1, 2], and in crises such as wars or natural disasters they face greater risks to survival and access to basic needs [3]. The United Nations (UN) Convention on the Rights of Persons with Disabilities (CRPD), ratified by nearly all countries, provides a universal ethical and legal framework for protecting these rights [4]. Upholding these principles during global crises is not a secondary task but a moral and scientific responsibility, as reaffirmed by the UN and other international organizations [5, 6, 7]. As Saraceno noted, promoting human rights requires not only advocacy but also the implementation of good practices within everyday professional contexts [8].

In line with this vision, our group has developed a mental health care model grounded in the CRPD and the World Health Organization (WHO) QualityRights framework [9, 10], which together represent the most comprehensive and operational synthesis of the rights-based approach to mental health. The CRPD reframes disability as the result of the interaction between impairments and social barriers, integrating the medical and social models into a unified paradigm. This framework positions persons with psychosocial disabilities as active decision-makers and rights-holders rather than passive recipients of care. The WHO QualityRights initiative operationalizes these principles into measurable objectives, promoting self-determination, improving care quality, developing community-based alternatives to institutionalization, empowering civil society, and aligning national legislation with the CRPD [9, 10, 11, 12, 13, 14, 15].

Importantly, the framework establishes a reproducible methodology for assessing and improving service quality, training professionals, and empowering users, thus offering both ethical guidance and practical tools for recovery-oriented and coercion-free care.

In line with biopsychosocial and rights-based perspectives, we also proposed an evolutionary model of mental disorders, particularly mood disorders, that conceptualizes these conditions as the outcome of traits that may be adaptive in specific contexts [16]. This approach moves beyond a strictly biomedical framework and situates individual functioning within a broader, dynamic interaction with the environment [17]. Such a systemic perspective requires overcoming medical reductionism and supports strategies that mitigate stigma and labeling. Furthermore, since 2015, our team in Cagliari has collaborated with the WHO and the European Union on QualityRights projects, going beyond the Italian research context [18], and has supported rights-based reforms in Ghana, Lebanon, Armenia, and Tunisia [19, 20, 21, 22, 23, 24].

These initiatives trained thousands of stakeholders, including traditional healers and service users, and fostered cross-cultural learning among professionals, researchers, and advocacy organizations. They demonstrated how international cooperation can enhance local capacity to implement coercion-free, community-oriented care even under conditions of political and economic instability.

Italy has long been regarded as a model for deinstitutionalization following the Basaglia law [25], yet in recent years the proportion of health expenditure devoted to mental health has declined from 5% in 2009 to below 3% after the pandemic [26]. This reduction jeopardizes community-based services and reflects a broader moral crisis concerning the rights of vulnerable minorities [27]. Within this context, our consultation-liaison psychiatry unit in Cagliari operates as a non-residential service integrating pharmacological, psychological, and rehabilitative interventions for persons with multiple diagnoses. The center promotes users’ active participation in therapeutic and life decisions and builds social inclusion networks with municipalities, Non-Governmental Organizations (NGOs), and cultural institutions. Despite positive experiences, such as community projects and evidence-based psychosocial interventions [28], control-oriented care cultures and insufficient resources remain key barriers to realizing a fully rights-based model.

Italy’s historical commitment to community psychiatry offers a unique opportunity to advance coercion-free, recovery-oriented mental health services. However, the translation of the CRPD–QualityRights framework into consultation-liaison psychiatry within general hospitals remains limited, and there is little empirical guidance on actionable priorities for implementation. To address this gap, we conducted an exploratory two-round Delphi study to elicit and compare expert views on how to operationalize recovery-oriented, human-rights-based practices in mental health care, with a particular focus on consultation-liaison psychiatry within a general hospital context. We adopted a Delphi variant suited to ethically and contextually complex domains, not to impose agreement but to surface both convergence and meaningful divergence, thereby integrating professional, experiential, and cultural knowledge from diverse settings and capturing latent consensus without forcing alignment [29]. Within this rationale, our objectives were to: (i) identify perceived benefits and risks of linking national work with international collaboration for rights-based reform; (ii) prioritize concrete actions to ensure human rights and promote recovery; (iii) highlight center-level practices that strengthen self-determination and social inclusion; and (iv) outline practical priorities for developing a coercion-free service and envisioning its future configuration in the Italian context. This approach provides focused, context-sensitive guidance for implementing the CRPD–QualityRights framework within hospital-based liaison services.

## 2. Materials and Methods

### 2.1 Study Design

This study employed an exploratory Delphi design consistent with recent methodological variants described in health sciences research [29]. Following the rationale of the policy Delphi approach [30], the study was conceived as an analytical exercise to elicit both convergence and dissensus among a heterogeneous panel of experts on complex, value-laden issues. In line with the interpretation of healthcare systems as complex adaptive systems, where linear and consensus-driven approaches may overlook contextual interdependencies [31], this design sought to explore the spectrum of expert perspectives rather than to impose agreement.

In line with contemporary interpretations of the Delphi as an adaptive and reflexive inquiry process [29, 30], this study was guided by the following methodological principles:

• Exploratory purpose, not consensus-oriented: no a priori agreement threshold was set, as the aim was to map both convergence and dissensus.

• Intentional heterogeneity of the panel: experts were selected for their diverse disciplinary, experiential, and geographical backgrounds.

• Iterative two-round structure: an open-ended first round followed by a structured second round with controlled feedback.

• Participant anonymity: responses were collected individually via e-mail to reduce dominance bias.

• Descriptive analytic focus: results were interpreted in terms of thematic salience rather than consensus metrics.

Consistent with this framework, a two-round design was considered methodologically appropriate for a Policy Delphi, whose purpose is to structure reflection and elicit informed divergence rather than to pursue iterative convergence. For this reason, additional rounds, typical of consensus-oriented Delphi variants, were not required.

In this context, the Delphi method functioned primarily as an analytical and reflective tool to map expert reasoning and to generate conceptually saturated themes that could inform future operational guidelines. This structure allowed the integration of professional, experiential, and cultural knowledge across disciplines and geographical contexts, while preserving the diversity of perspectives and avoiding premature alignment around any dominant narrative.

### 2.2 Expert Panel

Consistent with the Policy Delphi rationale, the expert panel was composed to capture a broad spectrum of informed perspectives on rights-based mental health [32]. Seventeen participants were invited through purposive sampling and contacted via e-mail. All were fluent in English, which served as the working language. Selection criteria emphasized diversity in disciplinary background, professional expertise, and geographical and socio-cultural context, key features that enhance the interpretive richness of Policy Delphi exercises [30, 32]. The panel included professionals with recognized expertise in mental health, psychosocial rehabilitation, primary care and human rights, as well as service users, researchers and activists in the field. Primary care professionals were included because they represent the first point of contact for emerging mental health needs and a key component of community-based pathways [33, 34]. This allowed the panel to encompass the full spectrum of clinical and contextual perspectives, from initial access to highly specialised psychosocial practice.

This composition reflected the cross-sectoral and participatory nature of human rights work in mental health and supported the examination of differing standpoints rather than convergence alone. Although grounded primarily in the Italian context, the inclusion of experts from multiple countries allowed identification of points of conceptual resonance across diverse policy environments. To mitigate contextual disparities, the questionnaire was formulated to be conceptually clear, and clarifications were provided when needed. Participants were blind to each other’s identities during both rounds, consistent with the Delphi principle of anonymity aimed at minimizing dominance bias and fostering independent reflection.

All nine experts who completed both rounds had substantial professional experience, typically more than ten years, and/or had contributed to the scientific and policy debate through relevant research, publications, or project involvement. Of the seventeen invited experts, nine completed both rounds, as summarised in Table 1.

**Table 1.** **Description of the expert panel**.

Participant	Country	Gender	Area(s) of expertise	Affiliation
P1	North Macedonia	M	Mental health	National Alliance for Neuromuscular Diseases and Neuroscience (GANGLION), Skopje
P2	Brazil	M	Mental health	Institute of Psychiatry, Federal University of Rio de Janeiro
P3	Armenia	F	Mental health	Armenian Psychiatric Association, Yerevan
P4	Nigeria	M	Mental health	Dept. of Psychiatry, University of Ibadan/Stellenbosch University
P5	Germany	M	Primary care	Dept. of General Practice, University Hospital Würzburg
P6	Italy	F	Advocacy in mental health	ASARP; University Hospital of Cagliari
P7	Lebanon	F	Medical anthropology, advocacy in mental health	Mental Health Service User Association, Beirut/University of Cagliari
P8	Colombia	M	Mental health, primary care	PhD Program in Tropical Medicine, Universidad Popular del Cesar
P9	Tunisia	F	Mental health	Razi Hospital, Tunis

ASARP, Associazione Sarda per l’Attuazione della Riforma Psichiatrica; M, male; F, female; Dept, department.

### 2.3 Procedure

The study was conducted between December 2024 and May 2025. The number of rounds was predetermined at two. Up to three reminders were sent to non-respondents three weeks intervals during each round, and their responses were collected via e-mail.

#### 2.3.1 Round 1 - Open-Ended Questionnaire

In the first round, participants received an open-ended questionnaire with four exploratory questions addressing the foundational dimensions of a rights-based approach in mental health. Responses were limited to a maximum of ten lines per question. Specifically, participants were invited to reflect on the following topics, in line with the aim of the study, as detailed in Appendix 1:

• The perceived benefits of combining national and international strategies;

• Key actions to promote human rights and recovery;

• Positive practices or structural elements observed at the Cagliari consultation-liaison center;

• Recommendations to strengthen rights-based practices in similar settings.

Participants’ responses were collected and analyzed qualitatively in order to develop the items for the second round of the procedure.

#### 2.3.2 Round 1 - Data Analysis 

Open-text responses from the first round were analyzed using inductive thematic analysis, with the aim of identifying conceptually saturated themes and organizing them into a coherent structure.

An initial coding of the data was independently conducted by two members of the research team, who reviewed all responses question by question. Emerging codes were discussed collaboratively, and overlapping or redundant categories were consolidated. The analysis prioritized conceptual clarity, consistency across questions, and fidelity to the language and perspectives of participants.

#### 2.3.3 Analytic Approach and Codebook Development

An inductive, semantic thematic analysis was conducted following Braun and Clarke’s six phases (familiarization; initial coding; theme searching; theme review; theme definition/naming; reporting) [35]. Two researchers independently read all responses end-to-end and wrote analytic memos; generated line-by-line descriptive codes; and collated codes into candidate themes at the semantic level. A codebook was then produced that specified for each code: label and definition. Disagreements were resolved through discussion; a third senior researcher adjudicated unresolved cases and audited the codebook to ensure conceptual clarity.

#### 2.3.4 From Codes to Themes

After iterative review, the team agreed on a final set of themes for each Round-1 question. Each theme included: (a) a concise definition; (b) inclusion/exclusion notes; and (c) 1–2 verbatim quotations selected for clarity and representativeness. Frequencies were tabulated only to enhance transparency (e.g., “theme mentioned by ~⅔ of respondents”), not as tests of generalizability. This process resulted in a finalized codebook documenting the thematic structure of Round-1 data (see Table 2 for an example). These themes formed the conceptual foundation for the second-round questionnaire, ensuring that each item was empirically grounded in participants’ qualitative input.

**Table 2.** **Example of codebook structure for Round-1 thematic analysis**.

Code/Theme label	Definition	Include when…	Exclude when…	Illustrative quote
Mutual exchange & learning	International collaboration as reciprocal sharing of practices and capacity building	The text stresses two-way learning, peer exchange, co-construction	Purely one-way transfer or compliance to standards	“Cooperating on an international level leads to the sharing of capacities between partners, including knowledge and technical skills.”

#### 2.3.5 From Themes to Round-2 Items: Controlled Feedback Procedure

Building on the finalized codebook, Round-1 themes were translated into closed-ended items (see Appendix 2) for Round-2 through a controlled feedback procedure:

• Theme distillation: for each question, we selected the most saturated and conceptually distinct themes, excluding marginal ideas while preserving diverse perspectives, especially those grounded in direct practice;

• Operational wording: we drafted clear and mutually exclusive options reflecting those themes, avoiding redundancy and double-barrelled phrasing;

• Prioritization constraint: a single-choice format was used to elicit relative salience rather than consensus;

• Clarity and consistency check: the research team reviewed items for comprehensibility and semantic independence, ensuring consistency with participants’ original wording (semantic fidelity).

This process resulted in eight structured items for Round-2, each corresponding to a distinct and data-grounded theme derived from Round-1 analysis.

#### 2.3.6 Round 2 - Structured Survey

Based on the analysis of the first round, a structured questionnaire was developed for round 2 (see Appendix 2), with the strategic aim not merely to confirm previously expressed views, but rather to stimulate critical comparison and promote more operational and focused reflection among participants. Specifically, the questionnaire was designed:

• To translate the most saturated and recurring themes into clear, distinct, and mutually exclusive response options;

• To encourage prioritization, ask participants to select only one option per item;

• To avoid vague or redundant formulations, thereby improving specificity and interpretability.

Consistent with the aim of mapping a plurality of positions rather than driving convergence toward consensus, we designed the second round using forced-choice items, in which each panellist was asked to indicate a single top priority. This choice is methodologically justified as an intra-respondent prioritisation device rather than as a consensus mechanism. Adding an ipsative/forced-choice phase has been shown to elicit clearer and sometimes counterintuitive priority structures [36], and forced-choice responses nonetheless appear to yield valid and reliable evaluations of quality [37]. Used in this way, forced-choice questioning clarifies which option each expert would back if compelled to choose, while analysis of the overall distribution of selected priorities preserves the plurality of perspectives sought in a policy Delphi. In line with this rationale, the second-round questionnaire consisted of eight multiple-choice items, each offering 5–6 mutually exclusive options. Participants selected only one option per item, thereby operationalising the forced-choice prioritisation approach described above.

#### 2.3.7 Round 2 - Data Analysis 

Quantitative data from round 2 were analyzed using descriptive statistics. For each item, the number and percentage of selections were calculated. Options receiving the highest selection frequencies were interpreted as signals of greater salience (majority preferences) rather than consensus, with particular attention paid to items consistently ignored or receiving minimal support, as these too offered insights into what participants did not prioritize. Given the exploratory purpose of the study, the goal is to map diverse expert views and detect nuanced areas of convergence.

## 3. Results

### 3.1 Round 1 of the Delphi Survey

The first round of the Delphi study generated a substantial body of qualitative data, which was coded and grouped by question to facilitate systematic thematic extraction. This semantic process informed the design of the second-round questionnaire by highlighting the most salient and frequently recurring themes, while intentionally avoiding redundancy or inclusion of concepts that had already achieved broad convergence, in order to reflect key ideas and to prompt prioritization and elicit more granular responses in the subsequent phases.

#### 3.1.1 Question 1

Original prompt: “What benefits can working both on a national level and through international projects bring to promoting and implementing a human rights-based approach in (mental) health services?”

Most respondents emphasized the value of integrating national and international levels to strengthen human rights-based mental health care. The most saturated theme (identified in approximately two-thirds of responses) was the ability to adapt global models to national contexts. This was seen as a way to enhance reform efforts and overcome systemic limitations. A majority also noted the importance of international cooperation in facilitating the exchange of good practices and mutual learning. Several participants highlighted how international frameworks can lend political legitimacy to rights-based reforms, making them harder to marginalize. Themes with medium saturation included: the role of international collaboration in training health professionals; and, to a lesser extent, the empowerment of service users through exposure to international standards. Less frequently, participants warned against the risk of uncritical transfer of models across contexts.

These themes directly shaped the structured items in Round 2, which asked participants to distinguish between adaptive, pedagogical, and political benefits of international engagement.

#### 3.1.2 Question 2 

Original prompt: “What key actions or measures do you believe are essential to implement in mental health services to ensure human rights and promote a recovery-based approach?”

There was strong semantic convergence around the need for systemic change in both service organization and daily practices. The most saturated themes (appearing in over 75% of responses) were: the active involvement of persons with psychosocial disabilities in both governance and individual care planning; and the need to strengthen training for professionals on human rights, shared decision-making, and trauma-informed care. A significant number of respondents also stressed the importance of shifting from custodial to community-based models of care. Themes with medium saturation included efforts to reduce cultural, economic, and geographical barriers to access. Less commonly, respondents mentioned the need to monitor human rights implementation and to integrate mental health into broader public policies. These findings informed Round 2 items asking participants to prioritize among participation, training, accessibility, and service transformation.

#### 3.1.3 Question 3 

Original prompt: “Which specific aspects identified during your visits to our center in Cagliari (Italy) do you consider most crucial for promoting self-determination, awareness of rights and social inclusion of persons with psychosocial disabilities?”

Respondents consistently highlighted the relational dimension of care observed at the Cagliari center. The most saturated theme (appearing in approximately 75% of responses) was the emphasis on a person-centered, non-hierarchical relationship between users and staff-based on active listening, flexibility, and respect. Roughly half of the participants praised the center’s commitment to promoting self-determination, including mechanisms for shared decision-making and meaningful participation. Several also noted the center’s connection with community resources as an important driver of social inclusion. Themes with medium saturation included the welcoming, informal atmosphere and the ongoing training of staff. Marginal themes included the use of expressive and artistic media. These observations informed Round 2 questions focusing on practical strategies to support self-determination and the professional roles involved in fostering rights-based practices.

#### 3.1.4 Question 4 

Original prompt: “What recommendations would you suggest for implementing in our center in Cagliari in order to improve the approach for promoting the rights of individuals with psychosocial disabilities?”

Responses fell into three main categories, which were reflected in the second-round questionnaire: strengthening community orientation, diversifying therapeutic approaches, and improving service accessibility. Two-thirds of participants recommended reinforcing connections with local communities through collaboration with associations, social services, and informal networks. Half of the experts emphasized the need to expand available therapeutic options, including greater involvement of peer-supporters and recognition of experiential knowledge. A third of the responses focused on making the center more accessible, particularly through digital tools and outreach strategies to rural or underserved populations. Themes with medium saturation included cultural sensitivity training and the use of multilingual tools. These themes were translated into structured items in Round 2, allowing participants to prioritize among community engagement, accessibility, diversity of approaches, and intercultural competence.

### 3.2 Round 2 of the Delphi Survey

#### 3.2.1 Question 1 

“What is the main benefit of international collaboration in promoting human rights in mental health services?”

During the second round of the Delphi survey, participants were asked to identify what they considered to be the primary benefit of international collaboration in advancing human rights within mental health services. A clear majority (78%) selected “mutual exchange and learning”, emphasizing the value of reciprocal sharing of good practices and capacity building across national contexts. This may reflect a vision of international cooperation as a horizontal and dialogical process, where knowledge and innovation are co-constructed rather than transferred unidirectionally. In contrast, “local adaptation of global strategies” was chosen by only two participants (22%). While this indicates that contextual implementation is still valued, it appears to be perceived as a secondary, operational aspect, rather than the core benefit of international engagement. Notably, none of the participants selected options related to access to economic or technical resources, policy alignment with international frameworks (e.g., CRPD), increased user participation in policymaking, or professional training on human rights. This lack of convergence around more instrumental or structural dimensions suggests that respondents may perceive international collaboration as most valuable when it fosters mutual empowerment and collective development, rather than when it provides predefined resources, policy frameworks, or training initiatives. This perspective may imply a preference for a model of collaboration that prioritizes co-learning and the exchange of practices over top-down standardization or technical assistance, which may be perceived as prescriptive and externally imposed aspects of international partnerships, as shown in Table 3.

**Table 3.** **Participant responses to question 1**.

Option	Responses	% (n = 9)
Mutual exchange and learning	7	78%
Local adaptation of global strategies	2	22%
Access to resources and funding	0	0%
Improvement of national policies	0	0%
Greater user involvement	0	0%
Training on human rights and mental health	0	0%

#### 3.2.2 Question 2 

“What do you consider the main limitation or risk of international collaboration in promoting mental health service reform?”

In the second question, participants were asked to identify the most significant limitation or risk associated with international collaboration in the reform of mental health services. The option selected most frequently was “lack of adaptation to available resources”, chosen by 5 out of 9 respondents (56%). This concern indicates the perception that internationally proposed reforms may be economically or organizationally unsuitable for local health systems, highlighting a need for feasibility and sustainability in international collaboration. The second most cited risk was the “risk of cultural imposition”, indicated by 3 participants (33%). This response points to apprehension that imported models may fail to respect local cultural, social, and economic contexts, potentially undermining both their acceptability and their effectiveness. Only 1 participant (11%) selected “local and institutional resistance” as the primary risk, while none of the respondents chose the remaining three options: loss of national control, excessive emphasis on foreign success models, or idealization of foreign models. These results, summarized in Table 4, suggest that participants may be less concerned with political or ideological risks (such as loss of autonomy or idealization of the “foreign”) and more attentive to contextual mismatches that could compromise the relevance or applicability of imported reforms. The low selection of resistance-related or nationalistic arguments indicates that opposition to international collaboration is not rooted in protectionism, but rather in the pragmatic need for contextual alignment. In summary, the findings underscore a view of international collaboration that must remain flexible, adaptive, and locally responsive, rather than standardized or prescriptive in nature. For these experts, the success of transnational partnerships in mental health reform depends on their ability to respect and work within local constraints, both systemic and cultural.

**Table 4.** **Participant responses to question 2**.

Option	Responses	% (n = 9)
Lack of adaptation to available resources	5	56%
Risk of cultural imposition	3	33%
Local and institutional resistance	1	11%
Loss of national control	0	0%
Excessive emphasis on foreign success models	0	0%
Idealization of foreign models	0	0%

#### 3.2.3 Question 3 

“What action do you consider a priority to ensure human rights and promote recovery in mental health services?”

The most frequently selected option was “social inclusion”, chosen by 4 out of 9 respondents (44%). This indicates a strong emphasis on access to employment, housing, and education as foundational conditions for autonomy and psychosocial well-being. The second most selected priority was “staff training”, indicated by 3 participants (33%). This underscores the recognition that mental health professionals require targeted education on human rights and recovery-oriented practices to effectively shift service culture toward more rights-based and recovery-oriented approaches. “Active user involvement” was chosen by 2 respondents (22%), highlighting the relevance of user participation in service design and decision-making, though this was not identified as the top priority. None of the participants selected the following options: Legislative reforms, Rights monitoring, or Recovery tools. The distribution of responses is reported in Table 5. These results suggest that participants tend to prioritize concrete, structural conditions (such as inclusion and professional education) over legal or procedural mechanisms for change. The lack of selection for legislative reform or monitoring implies that, while important, these may be viewed as secondary or supportive measures, insufficient on their own to promote recovery and rights in a meaningful way. In summary, the responses reveal a shared belief that ensuring rights and recovery in mental health requires addressing social determinants and empowering professionals, rather than relying solely on top-down reforms or tools.

**Table 5.** **Participant responses to question 3**.

Option	Responses	% (n = 9)
Social inclusion	4	44%
Staff training	3	33%
Active user involvement	2	22%
Legislative reforms	0	0%
Rights monitoring	0	0%
Recovery tools	0	0%

#### 3.2.4 Question 4 

“How can the decision-making power of users in mental health services be strengthened?”

The responses were evenly distributed across several options, suggesting multiple complementary pathways are considered important for user empowerment, as shown in Table 6. Two options were selected most frequently, each by 3 out of 9 participants (33%): “Decision support tools”—including recovery plans and structured supports to help users articulate their preferences and “Role in governance”—involving users in the design and management of services and policy-making. These selections suggest that respondents place equal value on individual-level autonomy-enhancing instruments and systemic user participation in governance. This pattern suggests a dual-level empowerment strategy, combining personal agency with structural influence. Following these, “Access to information” was selected by 2 participants (22%); “Overcoming paternalism”, referring to a cultural shift in clinical practice to ensure users are actively involved in therapeutic decisions, chosen by 1 respondent (11%) and “Peer support” that did not receive any votes. These findings suggest that participants favor structural and procedural changes, such as governance roles and decision-support mechanisms, over purely cultural or peer-led approaches. The absence of votes for peer support may reflect its limited institutional integration or a perception that it is less effective without systemic recognition and structure. In conclusion, participants endorse a multifaceted model of empowerment, one that combines access to tools and information with the opportunity to shape services at a governance level. While cultural transformation (e.g., overcoming paternalism) is acknowledged, it appears secondary to more tangible and systemically embedded mechanisms.

**Table 6.** **Participant responses to question 4**.

Option	Responses	% (n = 9)
Decision support tools	3	33%
Role in governance	3	33%
Access to information	2	22%
Overcoming paternalism	1	11%
Peer support	0	0%

#### 3.2.5 Question 5 

“Which health promotion strategy is most effective in fighting stigma?”

Reflecting a diversity of perspectives on anti-stigma strategies, the responses reveal a clear preference for community-based and educational interventions, with a wide distribution across several categories. The most frequently selected option was “Local communities—Social and work inclusion projects”, chosen by 4 out of 9 participants (44%). This reflects a shared belief in the transformative potential of direct inclusion in social and occupational environments as a means to normalize and destigmatize mental health conditions. Two other options were selected by 2 participants each (22%): “Media and institutions”—emphasizing the role of public campaigns and documentaries in raising awareness and “Schools and universities”—promoting the integration of mental health education into curricula from an early age. Only 1 participant (11%) selected: “Users as leaders”—involving people with lived experience as the primary agents of anti-stigma campaigns. Notably, no participant selected “Healthcare workers—Mandatory training to eliminate stigma within services”. The distribution of responses is reported in Table 7. These findings suggest that participants perceive community-level interventions, particularly those that enable social and professional inclusion, as the most effective long-term approach to combating stigma. Educational and media strategies are also seen as valuable, but somewhat secondary in impact. The low endorsement of user-led initiatives and the absence of support for professional training may point to a perception that stigma is rooted in broader societal structures, rather than primarily within services or among professionals. Alternatively, this could reflect skepticism about the efficacy or current institutional support for these approaches. In conclusion, the responses highlight a preference for structural, community-based strategies while institutional campaigns and user-led efforts were endorsed less frequently, indicating that anti-stigma work is most effective when embedded in everyday environments that promote participation and inclusion.

**Table 7.** **Participant responses to question 5**.

Option	Responses	% (n = 9)
Local communities	4	44%
Media and institutions	2	22%
Schools and universities	2	22%
Users as leaders	1	11%
Healthcare workers	0	0%

#### 3.2.6 Question 6 

“What is the main barrier within services to promoting the self-determination of persons with psychosocial disabilities?”

The responses reflect a relatively balanced distribution across different types of structural and cultural limitations, as shown in Table 8, suggesting that barriers are perceived as multifaceted and interrelated. The most frequently selected response was: “Control-oriented approach”—described as a clinical culture more focused on protection than on autonomy—chosen by 3 out of 9 participants (33%). This reflects a critical view of the underlying philosophy of care, where users may be subject to paternalistic dynamics that inhibit autonomy. Two other options were selected by 2 participants each (22%): “Insufficient training”—indicating a lack of staff competence regarding human rights and autonomy-oriented practices—and “Few practical tools”—such as the absence of recovery plans or decision-making supports. Another 2 respondents (22%) chose: “Institutional barriers”—referring to rigid regulations and limited resources. No participant selected: “Lack of a dedicated role”—i.e., the absence of a professional figure explicitly tasked with supporting autonomy.

**Table 8.** **Participant responses to question 6**.

Option	Responses	% (n = 9)
Control-oriented approach	3	33%
Insufficient training	2	22%
Few practical tools	2	22%
Institutional barriers	2	22%
Lack of a dedicated role	0	0%

The relatively even distribution of responses reveals a shared understanding that barriers to self-determination are both systemic and operational. The most frequently identified issue, the control-oriented model of care, suggests that cultural transformation is seen as a precondition for advancing rights-based practices. However, the simultaneous emphasis on training, tools, and regulations implies that changing professional attitudes is not sufficient on its own. The absence of support for the creation of a dedicated professional role may indicate that participants see self-determination not as the task of a single figure, but as a responsibility to be integrated across the entire service system, or as something that does not require the support of a specific operator, but rather arises from contextual conditions or from the will of the individual user. In summary, these findings suggest that promoting self-determination requires multi-level interventions, addressing institutional rigidity, professional development, resource availability, and, most importantly, a shift in the foundational ethos of care from control to autonomy.

#### 3.2.7 Question 7 

“What should be a further priority for a coercion-free center like ours?”

Reflecting a diversity of priorities, participants identified three options as additional strategic priorities for a coercion-free mental health center, each selected by 2 out of 9 respondents (22%): “Active user involvement”—highlighting the importance of including service users in decision-making processes and governance; “Social and work inclusion”—referring to partnerships with companies and communities to foster social participation and “Monitoring of quality and rights compliance”—including independent audits and structured user feedback. The full distribution of responses is presented in Table 9. “Inclusive cultural approach”—cultural mediators and practices adapted to the beliefs and values of the community; “Facilitated access to services”—through telemedicine, digital tools, and spatial accessibility and “Diversified therapeutic approaches”—the integration of holistic and innovative methods that respect individual preferences each received 1 vote (11%). The absence of a dominant response suggests that participants view the development of coercion-free services as a multifaceted effort, where diverse dimensions, cultural, organizational, and therapeutic, need to be addressed simultaneously. The equal prioritization of governance, inclusion, and quality monitoring indicates that respondents value user-centeredness, participation, and accountability as fundamental pillars of non-coercive practice. At the same time, the singular selections for culturally sensitive care, access, and therapeutic diversity highlight an awareness of specific operational levers that can enhance user experience and reduce the perceived need for coercion. In summary, the findings portray a shared orientation toward a pluralistic model of innovation, where no single intervention is sufficient, and priorities must be tailored to the specific needs, values, and social contexts of the people served.

**Table 9.** **Participant responses to question 7**.

Option	Responses	% (n = 9)
Active user involvement	2	22%
Social and work inclusion	2	22%
Monitoring of quality and rights compliance	2	22%
Inclusive cultural approach	1	11%
Facilitated access to services	1	11%
Diversified therapeutic approaches	1	11%

#### 3.2.8 Question 8 

“How do you imagine the mental health center of the future?”

In this final question, the responses reveal a clear orientation toward community-based, socially integrated care models, with limited interest in technocratic or institutional alternatives. As shown in Table 10, the most frequently selected option was: “Full community integration—Mental health connected with employment and social life”, chosen by 5 out of 9 participants (56%). This suggests a strong convergence around the idea that the future of mental health care lies in embedding support within everyday life contexts, promoting inclusion, and reducing isolation.

**Table 10.** **Participant responses to question 8**.

Option	Responses	% (n = 9)
Full community integration	5	56%
Center without walls	2	22%
Recovery budgets for users	2	22%
Management without traditional professionals	0	0%
Advanced technologies and AI	0	0%

AI, artificial intelligence.

The second most selected response was: “Center without walls”—Support through mobile teams, digital tools, and community spaces, selected by 2 participants (22%). This model emphasizes decentralization, flexibility, and accessibility, aligning with international trends in community mental health reform. Another 2 participants (22%) opted for: “Recovery budgets for users”—Freedom to choose care and services. This indicates support for user-directed models that prioritize autonomy and personalized care pathways. No participants selected: “Management without traditional professionals”—in which peer supporters take central roles. “Advanced technologies and AI”—including virtual reality or artificial intelligence applications. These results suggest that the future envisioned by participants is one where mental health care is fully embedded in the social fabric, closely linked to employment, relationships, education, and housing. The lack of support for purely technological or peer-led models may reflect skepticism toward disintermediation or an awareness of the institutional and cultural changes still needed for those innovations to be viable at scale. The fact that more than half of the panel chose community integration confirms a shift from the concept of the center as a place, toward one of function, connection, and presence within the community. In summary, the envisioned future mental health center is one that is inclusive, mobile, and relational, less reliant on physical infrastructure and more focused on supporting people in the spaces where life and recovery actually happen.

A synthesis of cross-cutting themes and response patterns is presented in Table 11. Several high-salience themes identified in Round 1—such as social inclusion, participatory governance, relational models of care, and attention to contextual adaptation—re-emerged in Round 2 as the most frequently selected options, indicating continuity in thematic salience across rounds. The Round 2 responses were reviewed across all eight items and grouped into broader macro-themes to capture recurring patterns of prioritization and areas of convergence among participants. A first consistent pattern concerns participatory and relational models. “Mutual exchange and learning” was identified as the main benefit of international collaboration (78%, Q1), and “full community integration” was the most frequent vision for future services (56%, Q8), with limited support for institutional or technological options across items.

**Table 11.** **Cross-cutting synthesis of findings across Delphi questions (Round 2)**.

Key theme	Main insight	Related questions
Participatory and relational models	Strong preference for mutual exchange and learning (78%) and full community integration (56%) over institutional or technical approaches.	Q1, Q3, Q5, Q8
Empowerment through shared governance	Equal support for governance roles and decision-support tools (33% each).	Q4
Skepticism toward top-down or standardized approaches	A majority identified lack of adaptation to available resources as the main concern of international collaboration (56%), followed by cultural imposition (33%). Resource-based, legislative, monitoring-based, and technology-driven options received no support. Mandatory training of healthcare workers was also not endorsed.	Q1, Q2, Q3, Q5, Q6, Q8
Multifactorial view of barriers	Responses to barriers were distributed across multiple causes (33% control-oriented model; 22% each for insufficient training, tools, institutional rigidity).	Q6
Pluralistic vision for coercion-free care	Absence of a dominant priority: participants distributed their choices evenly across multiple strategies (22% each).	Q7
Social determinants as levers of change	Social inclusion emerged as central across multiple domains: recovery (44%), anti-stigma strategies (44%), and visions of future services focused on community integration (56%).	Q3, Q5, Q8

Q, question.

Empowerment through shared governance was also reflected in respondents’ selections. Equal priority was assigned to governance roles and decision-support tools (33% each, Q4), indicating interest in both structural and individual-level mechanisms for enhancing user decision-making.

Consistent indications of skepticism toward top-down or standardized approaches were observed. The most frequent limitation of international collaboration was “lack of adaptation to available resources” (56%, Q2), followed by “cultural imposition” (33%, Q2). Options related to legislation, monitoring, training-only approaches, or advanced technologies received no support.

Responses concerning barriers to self-determination reflected a multifactorial view of barriers (Q6): selections were distributed across control-oriented culture (33%), insufficient training (22%), lack of tools (22%), and institutional rigidity (22%).

Regarding priorities for coercion-free practice, respondents expressed a pluralistic vision for coercion-free care, with responses evenly distributed across several strategies and no single dominant option (Q7). Finally, social determinants as levers of change were reflected through the prominence of social inclusion across several domains. Social inclusion was the most frequently selected priority for promoting recovery and rights (44%, Q3) and the most selected anti-stigma strategy (44%, Q5), and it aligned with the preferred future vision of services centered on community integration (56%, Q8). Overall, the data portray a consistent preference for participatory, community-embedded and context-sensitive models rather than standardized or technocratic reforms. Across all questions, participants emphasized that respect for human rights cannot be separated from social inclusion, shared governance, and the everyday contexts in which people live and recover.

## 4. Discussion

The findings of this Policy Delphi study outline a coherent vision of rights-based mental health care, in which human rights are understood less as technical or procedural standards and more as relational, participatory, and context-dependent processes. Across the eight questions, experts consistently prioritized approaches grounded in collaboration, inclusion, and community presence. This orientation echoes contemporary recovery frameworks that emphasize participation, agency and meaningful connection with one’s social environment [38, 39], and is consistent with human rights perspectives emphasizing dignity, participation, and contextual relevance in mental health practice [40].

A first overarching pattern concerns the preference for participatory and relational models. International collaboration was valued primarily for reciprocal learning, and the mental health center of the future was envisioned as closely embedded in community life. These views align with recovery models centered on connectedness, citizenship and co-production [38, 39], and are coherent with WHO QualityRights, which positions autonomy and participation as core dimensions of service quality and human rights compliance [9]. Empowerment was conceptualised as both an individual and structural process. Decision-support tools and governance roles received comparable endorsement, suggesting that autonomy is most effectively strengthened when personal decision-making and institutional participation evolve together. This interpretation aligns with evidence indicating that shared decision-making and user involvement improve both agency and service responsiveness [41]. A second cross-cutting theme is skepticism toward top-down or standardized approaches. The main risk identified in international collaboration, insufficient adaptation to local resources, reinforces the widely documented finding that reforms lacking contextual fit rarely achieve sustainability or fidelity over time [42]. Similarly, respondents did not prioritise isolated or technocratic strategies such as legislative reform, rights monitoring, mandatory staff training, or peer support. This suggests a preference for approaches embedded in everyday practice and community systems rather than procedural or compliance-driven reforms, a perspective consistent with rights-oriented models emphasizing contextual appropriateness and participatory implementation [40]. Barriers to self-determination were understood as multidimensional and interdependent. Cultural, organisational, and operational obstacles, control-oriented practices, limited training, lack of tools, and institutional rigidity received comparable levels of endorsement. This reflects a broad consensus that self-determination depends on the interaction between organisational ethos, staff skills, and the availability of practical supports [43]. The absence of support for a dedicated professional role reinforces the principle that autonomy cannot be delegated to a single figure, but must permeate the entire service culture and structure.

Finally, social determinants emerged as central levers of change. Social inclusion was the most recurrently prioritized strategy across questions, underscoring that rights and recovery are inseparable from access to employment, housing, education, and community participation, findings consistent with both the Lancet Commission on Global Mental Health and Sustainable Development [44] and evidence on the role of social inclusion in mental health outcomes [45]. The preference for fully community-embedded models of care, rather than technological or institutional alternatives, aligns with contemporary proposals for integrated, place-based mental health systems [42].

Taken together, these patterns delineate not only a conceptual model but also a practical direction for service transformation. The convergence around participatory governance, contextual adaptation, autonomy-supporting tools, and social inclusion suggests a roadmap in which training becomes less focused on generic awareness and more oriented toward concrete competencies, such as managing coercion-risk situations, implementing shared decision-making, and facilitating recovery planning within multidisciplinary teams. At the organisational level, experts envision services where users and caregivers contribute to planning and evaluation through structured co-decision mechanisms, supported by routinely collected rights-based indicators that complement traditional clinical metrics. At the system level, partnerships that promote employment, housing stability, and community participation become essential components of non-coercive, rights-based care, alongside periodic rights audits ensuring that participatory and contextual principles are sustained over time.

The reflections offered by experts suggest that several elements present in the Cagliari service, such as relational practice, attention to autonomy in everyday interactions, and community linkage, may reflect broader currents in contemporary rights-based reform. Their appearance within the Delphi synthesis indicates that such practices are not unique to a single setting, but resonate with priorities that are increasingly shared across systems.

In this perspective, rights-based reform appears not as a linear progression driven by top-down directives, but as a dynamic and context-responsive process in which relational practices, community integration, and shared responsibility collectively shape sustainable and coercion-free mental health care.

### Limitations

As with most Delphi studies, this research presents several methodological limitations that should be acknowledged while not undermining the relevance of its findings. First, although the number of participants who completed both rounds was limited (n = 9), this is consistent with the exploratory aim of the study and the qualitative nature of the first phase. Despite the small sample size, the recurrence of certain themes across different profiles (e.g., professionals and service users) suggests a degree of convergence worth noting. Although the two-round structure was appropriate to the exploratory purpose of this Policy Delphi, a third round might have provided additional nuance by expanding points of disagreement. Nonetheless, the thematic consistency observed between Round 1 and Round 2 suggests that the core priority patterns were already sufficiently defined, and further rounds were unlikely to yield substantial additional insight. Second, the transition from open-ended responses in Round 1 to closed questions in Round 2 required the research team to exercise interpretative judgment in synthesizing and framing the items. While efforts were made to preserve conceptual richness and clarity, some nuances may have been lost in the process. Moreover, the composition of the panel was predominantly European and American, with limited representation from regions such as East or South Asia. This geographical imbalance may have influenced the range of cultural and systemic perspectives captured, although the international diversity within the panel still allowed for meaningful cross-contextual reflection. Future work could deepen analysis by comparing how interpretations of key concepts shift across cultural and institutional settings.

## 5. Conclusions

The results of this Delphi survey highlight key areas of convergence around community inclusion, user participation, and contextual adaptability, offering insights that can inform the future development of consultation-liaison services. Rather than prescribing fixed models, the findings point to the need for flexible, participatory frameworks that are responsive to both structural conditions and lived experience.

## Availability of Data and Materials

The data presented in this study are available upon request from the corresponding author. The data are not publicly available due to privacy and ethical issues. The manuscript does not contain any copyrighted material such as figures, tables, or extracts from previously published work. All data, analyses, and materials presented are entirely original and of new production, developed specifically for this study.

## Appendix

### Appendix 1

First Round — Delphi survey

1. Benefits of Dual-Level Approach. What benefits can working both on a national level and through international projects bring to promoting and implementing a human rights-based approach in (mental) health services?

2. Actions/Measures for Ensuring Human Rights and Promoting Recovery. What key actions or measures do you believe are essential to implement in mental health services to ensure human rights and promote a recovery-based approach?

3. Usefulness of Shared Experiences. Which specific aspects identified during your visits to our center in Cagliari (Italy) do you consider most crucial for promoting self-determination, awareness of rights and social inclusion of persons with psychosocial disabilities?

4. Recommendations for Improvement. What recommendations would you suggest for implementing in our center in Cagliari in order to improve the approach for promoting the rights of individuals with psychosocial disabilities?

### Appendix 2

Second Round — Delphi survey

What is the main benefit of international collaboration in promoting human rights in mental health services?

*Select only one option*

1. Local adaptation of global strategies (Better opportunities to customize and implement in national contexts models developed elsewhere).

2. Mutual exchange and learning (Sharing of good practices and capacity building among countries).

3. Access to resources and funding (Economic and technical support for reforms).

4. Improvement of national policies (Alignment with international standards, e.g., CRPD, and support from global advocacy).

5. Greater user involvement (Active participation in policy decisions).

6. Training on human rights and mental health (Training for healthcare professionals).

What do you consider the main limitation or risk of international collaboration in promoting mental health service reform?

*Select only one option*

1. Risk of cultural imposition — International models may not respect the cultural, social, and economic specificities of the countries involved.

2. Lack of adaptation to available resources – Some internationally proposed reforms may not be economically or organizationally sustainable for local health systems.

3. Loss of national control — Dependence on external funding and directives may limit the decision-making autonomy of local services.

4. Excessive emphasis on foreign success models — Assuming that strategies effective elsewhere will work everywhere, without proper customization.

5. Local and institutional resistance — National professionals and institutions may perceive collaboration as interference, hindering reforms.

6. Idealization of foreign models — Risk of creating the myth of a perfect “elsewhere”, leading to distrust in the local context and devaluation of national resources.

What action do you consider a priority to ensure human rights and promote recovery in mental health services?

*Select only one option*

1. Active user involvement — Participation in service design and decision-making.

2. Staff training — Education on human rights and recovery.

3. Legislative reforms — Elimination of coercive measures and alignment with international standards.

4. Social inclusion — Access to employment, housing, and education to support autonomy.

5. Rights monitoring — Independent audits and reporting mechanisms for users.

6. Recovery tools — Personalized plans and advanced decision-making support.

How can the decision-making power of users in mental health services be strengthened?

*Select only one option*

1. Overcoming paternalism — Ensuring users have an active role in therapeutic choices.

2. Decision support tools — Recovery plans and advanced support to express preferences.

3. Role in governance — Involving users in health policies and service management.

4. Peer support — Promoting mutual support to reduce dependence on professionals.

5. Access to information — Training staff and making rights more understandable for users.

Which health promotion strategy is most effective in fighting stigma?

*Select only one option*

1. Users as leaders — Campaigns led directly by people with psychosocial disabilities.

2. Media and institutions — Public awareness through commercials and documentaries.

3. Schools and universities — Mental health education integrated into curricula.

4. Healthcare workers — Mandatory training to eliminate stigma within services.

5. Local communities — Social and work inclusion projects.

What is the main barrier within services to promoting the self-determination of persons with psychosocial disabilities?

*Select only one option*

1. Lack of a dedicated role — No specific professional supporting rehabilitation and autonomy.

2. Insufficient training — Limited staff preparation on self-determination and human rights.

3. Control-oriented approach — A healthcare culture focused more on protection than on freedom.

4. Few practical tools — Absence of recovery plans and advanced decision-making support.

5. Institutional barriers — Rigid regulations and limited resources.

What should be a further priority for a coercion-free center like ours?

*Select only one option*

1. Inclusive cultural approach — Cultural mediators and therapeutic practices adapted to the beliefs and values of the communities served.

2. Active user involvement — Participation in decisions regarding service governance.

3. Facilitated access to services — Telemedicine, digitalization, and accessible spaces.

4. Diversified therapeutic approaches — Integration of holistic and innovative techniques to respect individual preferences.

5. Social and work inclusion — Partnerships with companies and communities.

6. Monitoring of quality and rights compliance — Independent audits and structured user feedback.

How do you imagine the mental health center of the future?

*Select only one option*

1. Center without walls — Support through mobile teams, digital tools, and community spaces.

2. Management without traditional professionals — Peer supporters as central figures.

3. Recovery budgets for users — Freedom to choose care and services.

4. Full community integration — Mental health connected with employment and social life.

5. Advanced technologies and AI — Virtual reality and artificial intelligence for well-being.
